# VSGs Expressed during Natural T. b. gambiense Infection Exhibit Extensive Sequence Divergence and a Subspecies-Specific Bias towards Type B N-Terminal Domains

**DOI:** 10.1128/mbio.02553-22

**Published:** 2022-11-10

**Authors:** Jaime So, Sarah Sudlow, Abeer Sayeed, Tanner Grudda, Stijn Deborggraeve, Dieudonné Mumba Ngoyi, Didier Kashiama Desamber, Bill Wickstead, Veerle Lejon, Monica R. Mugnier

**Affiliations:** a Department of Molecular Microbiology and Immunology, Johns Hopkins Bloomberg School of Public Health, Baltimore, Maryland, USA; b Department of Biomedical Sciences, Institute of Tropical Medicine, Antwerp, Belgium; c Department of Parasitology, Institut National de Recherche Biomédicale, Kinshasa, Democratic Republic of the Congo; d Programme Nationale de Lutte contre la Trypanosomiase Humaine Africaine (PNLTHA), Ministry of Health, Kinshasa, Democratic Republic of the Congo; e School of Life Sciences, Queen’s Medical Centre, University of Nottingham, Nottingham, United Kingdom; f UMR-177 Intertryp, Institut de Recherche pour le Développement, Centre de Coopération Internationale en Recherche Agronomique pour le Développement, University of Montpellier, Montpellier, France; Yale University School of Public Health

**Keywords:** *Trypanosoma*, antigenic variation, genomics, host-pathogen interactions, variant surface glycoprotein

## Abstract

Trypanosoma brucei gambiense is the primary causative agent of human African trypanosomiasis (HAT), a vector-borne disease endemic to West and Central Africa. The extracellular parasite evades antibody recognition within the host bloodstream by altering its variant surface glycoprotein (VSG) coat through a process of antigenic variation. The serological tests that are widely used to screen for HAT use VSG as one of the target antigens. However, the *VSGs* expressed during human infection have not been characterized. Here, we use VSG sequencing (VSG-seq) to analyze the *VSGs* expressed in the blood of patients infected with T. b. gambiense and compared them to VSG expression in Trypanosoma brucei rhodesiense infections in humans as well as Trypanosoma brucei brucei infections in mice. The 44 *VSGs* expressed during T. b. gambiense infection revealed a striking bias toward expression of type B N termini (82% of detected VSGs). This bias is specific to T. b. gambiense, which is unique among T. brucei subspecies in its chronic clinical presentation and anthroponotic nature. The expressed T. b. gambiense *VSGs* also share very little similarity to sequences from 36 T. b. gambiense whole-genome sequencing data sets, particularly in areas of the VSG protein exposed to host antibodies, suggesting the antigen repertoire is under strong selective pressure to diversify. Overall, this work demonstrates new features of antigenic variation in T. brucei *gambiense* and highlights the importance of understanding *VSG* repertoires in nature.

## INTRODUCTION

Human African trypanosomiasis (HAT) is caused by the protozoan parasite Trypanosoma brucei. T. brucei and its vector, the tsetse fly, are endemic to sub-Saharan Africa (1). There are two human-infective T. brucei subspecies: T. b. gambiense, which causes chronic infection in West and Central Africa (~98% of cases), and T. b. rhodesiense, which causes acute infection in East and Southern Africa (~2% of cases) (2, 3). In humans, infections progress from an early stage, usually marked by a fever and body aches, to a late stage, associated with severe neurological symptoms, that begins when the parasite crosses the blood-brain barrier (4). HAT is considered fatal without timely diagnosis and treatment. While around 50 million people are at risk of infection (3), the number of annual human infections has declined significantly in recent years, with only 864 cases reported in 2019 (5). The World Health Organization is working toward zero human transmissions of HAT caused by T. b. gambiense (gHAT) by 2030 (6). Case detection and treatment are an important component of current public health initiatives to control the disease.

Prospects for developing a vaccine are severely confounded by the ability of African trypanosomes to alter their surface antigens (7). As T. brucei persists extracellularly in blood, lymph, and tissue fluids, it is constantly exposed to host antibodies (8–11). The parasite periodically changes its dense variant surface glycoprotein (VSG) coat to evade immune recognition. This process, called antigenic variation, relies on a vast collection of thousands of VSG-encoding genes (12–15). T. brucei also continually expands the number of usable antigens by constructing mosaic *VSGs* through one or more recombination events between individual *VSG* genes (16, 17).

Although the *VSG* repertoire is enormous and potentially expanding, these variable proteins are the primary antigens used for serological screening for gHAT (there is currently no serological test for diagnosis of infection with *T. b. rhodesiense*). One VSG in particular, LiTat 1.3, has been identified as an antigen against which many gHAT patients have antibodies (18) and, thus, serves as the main target antigen in the primary serological screening tool for gHAT, the card agglutination test for trypanosomiasis (CATT/T. b. gambiense) (19). More recently developed rapid diagnostic tests use a combination of native LiTat 1.3 and another VSG, LiTat 1.5 (20, 21), or the combination of a VSG with the invariant surface glycoprotein ISG 65 (22).

Despite the widespread use of VSGs as antigens to screen for gHAT, little is known about how the large genomic repertoire of *VSGs* is used in natural infections; the number and diversity of *VSGs* expressed by wild parasite populations remain unknown. It is unclear whether VSG repertoires are evolving in the field, potentially affecting the sensitivity of serological tests that use VSG as an antigen. Notably, some T. b. gambiense strains lack the LiTat 1.3 gene entirely (23, 24). A study from our lab that evaluated VSG expression during experimental mouse infections by VSG sequencing (VSG-seq), a targeted RNA-sequencing method that identifies the *VSGs* expressed in a given population of T. brucei, revealed significant *VSG* diversity within parasite populations in each animal (25). This diversity suggested that the parasite’s genomic VSG repertoire might be insufficient to sustain a chronic infection, highlighting the potential importance of the recombination mechanisms that form new VSGs (12, 16).

Given the role of VSGs during infection and their importance in gHAT screening tests, a better understanding of *VSG* expression in nature could inform the development of improved screening tests while providing insight into the molecular mechanisms of antigenic variation. To our knowledge, only one study has investigated *VSG* expression in wild T. brucei isolates (26). For technical reasons, this study relied on RNA isolated from parasites passaged through small animals after collection from the natural host. As *VSG* expression may change during passage, the data obtained from these samples are somewhat difficult to interpret. To better understand the characteristics of antigenic variation in natural T. brucei infections, we sought to analyze *VSG* expression in T. brucei field isolates from which RNA was directly extracted.

In the present study, we used VSG-seq to analyze the *VSGs* expressed by T. b. gambiense in the blood of 12 patients with a confirmed infection. To complement these data, we also used our pipeline to analyze published transcriptome sequencing (RNA-seq) data sets from both experimental mouse infections and *T. b. rhodesiense* patients. In addition to VSG-seq, we searched for evidence of sequence homology in a large set of whole-genome sequences for a variety of T. b. gambiense isolates. Our analysis revealed distinct biases in *VSG* expression that appear to be unique to the T. b. gambiense subspecies and a divergence between expressed patient *VSGs* and previously characterized T. b. gambiense strains that suggests patient *VSG* repertoires are diversifying rapidly.

## RESULTS

### Parasites in gHAT patients express diverse sets of VSGs.

**FIG 1:**
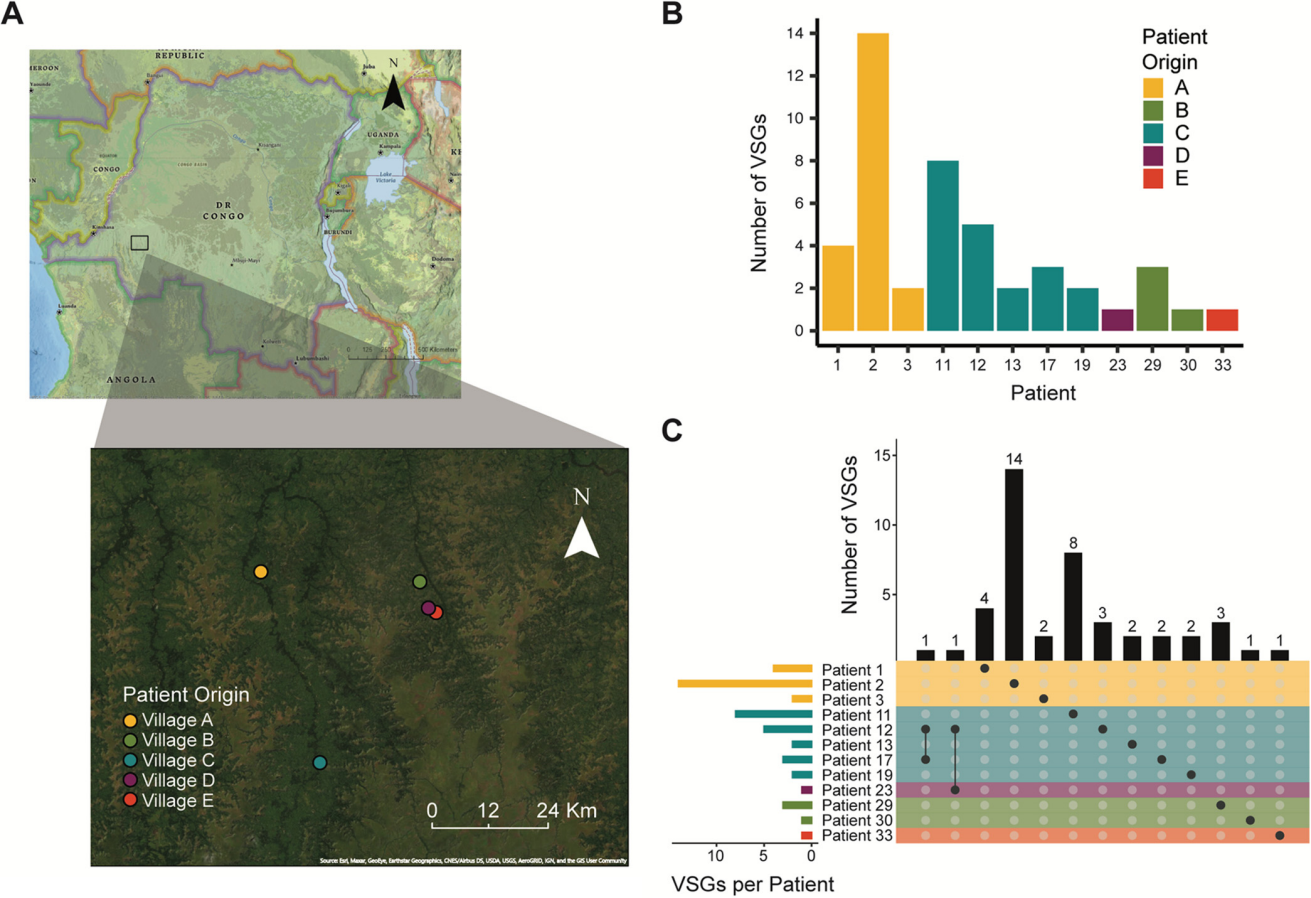
Parasites isolated from gHAT patients express multiple *VSGs*. (A) Map showing the location of each patient’s home village. Maps were generated with ArcGIS software by Esri using world imagery and National Geographic style basemaps. (B) Graph depicting the total number of *VSGs* expressed in each patient. (C) The intersection of expressed *VSG* sets in each patient. Bars on the left represent the size of the total set of VSGs expressed in each patient. Dots represent an intersection of sets with bars above the dots representing the size of the intersection. Color indicates patient origin.

To investigate *VSG* expression in natural human infections, we performed VSG-seq on RNA extracted from whole blood collected from 12 human African trypanosomiasis patients from five locations in the Kwilu province of the Democratic Republic of the Congo (DRC) (Fig. 1A). We estimated the relative parasitemia of each patient by Spliced-Leader quantitative PCR (SL-QPCR) (27), and we estimated the number of parasites after mini Anion Exchange Centrifugation Technique (mAECT) on buffy coat for all patients except patient 29 (Table 1). Using RNA extracted from 2.5 mL of whole blood from each patient, we amplified T. brucei RNA from host/parasite total RNA using a primer against the T. brucei spliced leader sequence and an anchored oligo-dT primer. The resulting trypanosome-enriched cDNA was used as a template to amplify *VSG* cDNA in three replicate reactions, and *VSG* amplicons were then submitted to VSG-seq sequencing and analysis. To determine whether a *VSG* was expressed within a patient, we applied the following stringent cutoffs. (i) We conservatively estimate that each 2.5-mL patient blood sample contained a minimum of 100 parasites. At this minimum parasitemia, a single parasite would represent 1% of the population (and consequently ~1% of the parasite RNA in a sample). As a result, we excluded all *VSGs* comprising <1% of the total VSG-seq pool in each patient as unlikely to represent the major expressed VSG in at least one cell from the population. (ii) We classified *VSGs* as expressed if they met the expression cutoff in at least two of three technical library replicates.

**TABLE 1:** Patient stage and parasitemia data^*a*^

Patient	Location (village)	Est. parasites in 500 μL buffy coat	Mean SL-RNA *C_T_*	WBC	Parasites in CSF	Stage
1	A	>50	22.155	1	−^*b*^	First
2	A	>50	19.020	6	−	Early second
3	A	2–5	28.780	6	−	Early second
11	C	>50	22.030	9	−	Early second
12	C	6–20	25.430	6	−	Early second
13	C	6–20	26.635	12	−	Early second
17	C	21–50	24.495	13	−	Early second
19	C	1	28.245	7	−	Early second
23	D	6–20	27.085	2	−	First
29	B		28.320	3	−	First
30	B	>50	22.960	694	+	Severe second
33	E	1	32.385	2	−	First

a We used the following staging definitions: first, 0 to 5 WBC/μL, no trypanosomes in cerebrospinal fluid (CSF); second, >5 WBC/μL or trypanosomes in CSF (with early 2nd defined as 6 to 20 WBC/μL and no trypanosomes in CSF and severe 2nd defined as >100 WBC/μL). WBC, white blood cells.

b The dashes in table indicate that patients were negative (−) by microscopy for parasites in the CSF, only one patient in the table was positive (+).

A total of 1,112 unique *VSG* open reading frames were assembled *de novo* from the patient reads, and 44 met our expression criteria. Only these 44 *VSGs*, which we will refer to as “expressed *VSGs*,” were considered in downstream analysis, except when otherwise noted. TgsGP, the VSG-like protein that partially enables resistance to human serum in T. b. gambiense (28), assembled in samples from patients 2, 11, 13, and 17 and met the expression threshold in patients 2, 11, and 17. The absence of this transcript in most samples is likely due to the low amount of input material used to prepare samples.

At least one *VSG* met our expression criteria in each patient, and in most cases, multiple *VSGs* were detected. Patient 2 showed the highest diversity, with 14 *VSGs* expressed (Fig. 1B; see also Fig. S1 in the supplemental material). There is a positive correlation between parasitemia, as estimated by quantitative PCR (qPCR), and the number of detected *VSGs* (see Fig. S2A in the supplemental material), suggesting that our blood volumes may not be sampling the full diversity of circulating expressed VSG at low parasitemia. Nevertheless, two *VSGs* were shared between patients as follows: *VSG* “Gambiense 195” was expressed in both patient 12 and patient 17 from village C and *VSG* “Gambiense 38” was expressed in patient 12 from village C and patient 23 from village D (Fig. 1C). Because our sampling did not reach saturation, resulting in some variability between technical replicates, we focused only on the presence/absence of individual VSGs for further analysis rather than relative expression levels within each population.

10.1128/mbio.02553-22.1 FIG S1 Heatmap of all assembled T. b. gambiense patient VSGs. Greyscale shows log_10_ of the estimated percentage of the parasite population expressing each VSG. Variants expressed by less than 1% of parasites considered not detected (n.d.). Download FIG S1, TIF file, 2.3 MB. Copyright © 2022 So et al. 2022 So et al. https://creativecommons.org/licenses/by/4.0/ This content is distributed under the terms of the Creative Commons Attribution 4.0 International license.

10.1128/mbio.02553-22.2 FIG S2 Correlation between parasitemia and diversity and N-terminal type distribution. (A) Correlation plots for T. b. gambiense-infected patients. (B) Correlation plots for *T. b. rhodesiense*-infected patients from Mulindwa et al. (35). (C) Correlation plots for VSG diversity and percent of N-terminal domain type B for *T.b. brucei*-infected mice from Mugnier et al. (25). Download FIG S2, EPS file, 2.0 MB. Copyright © 2022 So et al. 2022 So et al. https://creativecommons.org/licenses/by/4.0/ This content is distributed under the terms of the Creative Commons Attribution 4.0 International license.

### Natural T. b. gambiense infections show a strong bias toward the expression of type B VSG.

To further characterize the set of expressed *VSGs* in these samples, we sought to define the VSG domain types encoded by each *VSG.* T. brucei VSG contains two domains as follows: a variable N-terminal domain of ~350 to 400 amino acids and a less variable C-terminal domain of ~40 to 80 amino acids, characterized by one or two conserved groups of four disulfide-bonded cysteines (12, 29). On the surface of trypanosomes, the VSG N-terminal domain is readily exposed to the host. In contrast, the C-terminal domain is proximal to the plasma membrane and largely hidden from host antibodies (30–32). The N-terminal domain is classified into two types, A and B, each further distinguished into subtypes (A1-3 and B1-2), while the C-terminal domain has been classified into six types (1–5, 12, 29). These classifications are based on protein sequence patterns anchored by the conservation of cysteine residues, but the biological implications of VSG domain types have not been investigated.

**FIG 2:**
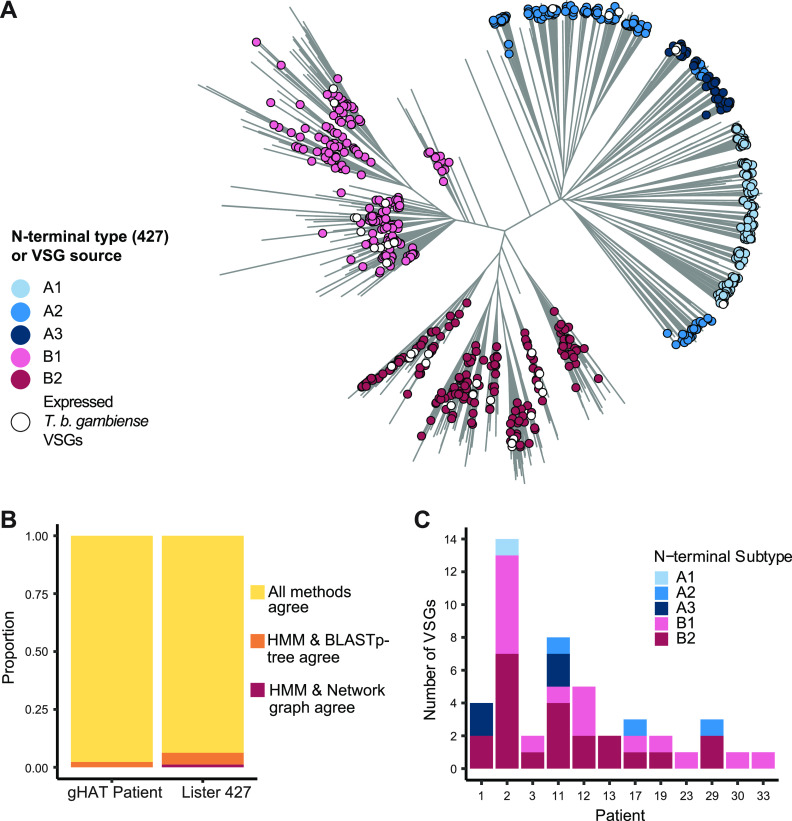
T. b. gambiense samples show a bias toward the expression of type B VSG. (A) Visualization of relatedness between N-terminal domain peptide sequences inferred by neighbor-joining based on normalized BLASTp scores. Legend indicates classification by HMM pipeline (for Lister 427 VSGs to highlight agreement between the two methods) or by subspecies for *VSGs* expressed in patients. (B) Agreement between three VSG typing methods for Lister 427 *VSG* set and the expressed T. b. gambiense patient *VSG* set. (C) N-terminal domain subtype composition of expressed T. b. gambiense *VSGs* as determined by HMM analysis pipeline.

We evaluated two automated approached for determining the type and subtype of each VSG’s N-terminal domain. The first approach was to create a bioinformatic pipeline to determine each N-terminal domain subtype, using hidden Markov model (HMM) profiles that we created for each subtype from sets of N-terminal domains previously typed by Cross et al. (14). The second approach was to create a BLASTp network graph based on a published method (33) where the N-terminal subtype of a VSG is determined by the set of VSGs it clusters with, and clusters are identified using the leading eigenvector method (34). We used each approach to determine the N-terminal subtype of each expressed *VSG* in our patient sample data set, along with 863 VSG N termini from the Lister 427 genome. We compared these results to either existing N-terminal classification (for Lister 427 VSGs) or classification based on position in a newly-generated BLASTp-tree (14) (for T. b. gambiense VSGs) (Fig. 2A, see also Fig. S4 in the supplemental material).

Both the new HMM profile and BLASTp network graph approaches generally recapitulated previous VSG classification based on BLASTp-tree, with all three methods agreeing 93.7% of the time (Fig. 2B). The HMM pipeline method agreed with BLASTp-tree typing for all patient VSGs, while the network graph approach agreed for 43/44 VSGs (Fig. 2B; see also Fig. S3 in the supplemental material) (14). It is not surprising that the HMM pipeline would better reflect the results of the BLASTp-tree method, as the N-terminal subtype HMM profiles were generated using VSGs classified by this method. Based on these data, we determined that the HMM method is a fast and accurate approach for determining the N-terminal domain types of unknown VSGs.

10.1128/mbio.02553-22.3 FIG S3 (A) Network plot showing peptide sequence relatedness between N-terminal domains. Each point represents a VSG N terminus. A link was drawn between points if the BLASTp E value was less than 10^−2^. Colors and shaded circles represent community assignments determined by the clustering algorithm. (B) The same graph as in panel A, but points are manually colored by known N-terminal subtype from Cross et al. (14) or by subspecies for VSGs identified in patients. Download FIG S3, TIF file, 1.3 MB. Copyright © 2022 So et al. 2022 So et al. https://creativecommons.org/licenses/by/4.0/ This content is distributed under the terms of the Creative Commons Attribution 4.0 International license.

Our N-terminal domain typing pipeline identified the domain sequence and subtype for all 44 patient VSGs (Fig. 2C). Of the expressed T. b. gambiense VSGs, 82% had type B N-terminal domains, and 50% or more of expressed *VSGs* within each patient were type B. This bias was not restricted to highly expressed *VSGs*, as 74.5% of all assembled *VSG* (813 of 1,091 classifiable to an N-terminal subtype) were also type B.

Using the network graph approach, we also tentatively assigned C-terminal domain types to the T. b. gambiense VSGs (see Fig. S5 in the supplemental material). In line with previous observations, we saw no evidence of domain exclusion: a C-terminal domain of one type could be paired with any type of N-terminal domain (Fig. S5E) (19). Most patient C-terminal domain types were type 2, while the remaining types were predominantly type 1, with only one type 3 C terminus identified in the patient set. Overall, these data suggest that, like N termini, expressed VSG C termini are also biased toward certain C-terminal types. Together, these observations motivated further investigation into the VSG domains expressed during infection by other T. brucei subspecies. We focused this analysis on expressed N-terminal domains, which make up most of the VSG protein, are more variable than C-terminal domains (14, 33), and are most likely to directly interface with the host immune system during infection (32).

10.1128/mbio.02553-22.4 FIG S4 BLASTp-tree of all T. b. gambiense *VSGs.* Download FIG S4, PDF file, 9.1 MB. Copyright © 2022 So et al. 2022 So et al. https://creativecommons.org/licenses/by/4.0/ This content is distributed under the terms of the Creative Commons Attribution 4.0 International license.

10.1128/mbio.02553-22.5 FIG S5 Expressed VSG C termini are primarily type 1 and type 2. (A) BLASTp-tree of C-terminal domains. Points are colored based on previously determined C-terminal type from Cross et al. (14) or by the source of the sequence (genomic or expressed) for T. b. gambiense *VSGs*. (B) Network plot showing peptide sequence relatedness between C-terminal domains in T. b. gambiense expressed VSGs. Each point represents a VSG C terminus. A link was drawn between points if the BLASTp E value was less than 1 × 10^−3^. Points are colored by the cluster determined by the clustering algorithm. Shaded circles also indicate clusters. (C) Same network plot as in panel B but colored by previously determined C-terminal type from Cross et al. (14) or by source for unclassified genomic or expressed *VSGs*. (D) VSG C-terminal types, based on cluster assignment visualized in panel B, in genomic and expressed *VSG* sets. (E) Pairing of C and N termini in T. b. gambiense patients. (F) C termini detected in each patient village. Download FIG S5, TIF file, 2.7 MB. Copyright © 2022 So et al. 2022 So et al. https://creativecommons.org/licenses/by/4.0/ This content is distributed under the terms of the Creative Commons Attribution 4.0 International license.

### Type B VSG bias is unique to T. b. gambiense infection.

**FIG 3:**
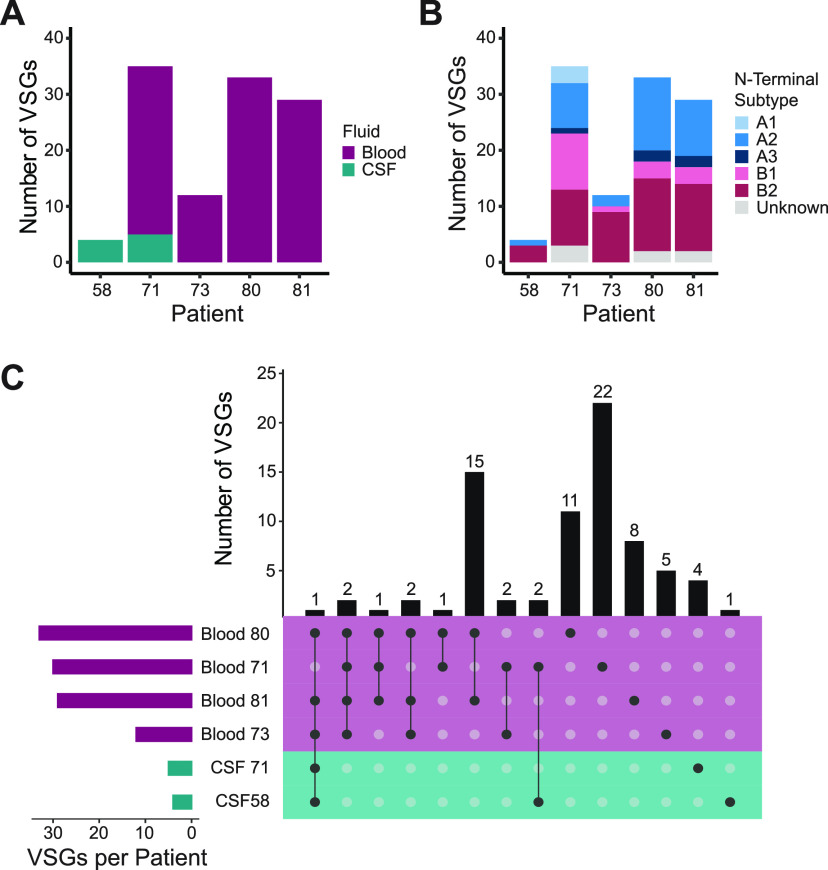
*T. b. rhodesiense* samples reveal diverse *VSG* expression but little N-terminal type bias. (A) The total number of expressed *T. b. rhodesiense VSGs* in each patient and sample type. Bar color represents the sample type from which RNA was extracted. (B) N-terminal domain subtype composition of all expressed *VSGs*. (C) Intersections of *VSGs* expressed in multiple infections. Bars on the left represent the size of the total set of VSGs expressed in each patient. Dots represent an intersection of sets, with bars above the dots representing the size of the intersection. Color indicates patient origin.

To determine whether the bias toward type B VSGs was specific to T. b. gambiense infections, we analyzed RNA-seq data from a published study measuring gene expression in the blood and cerebrospinal fluid (CSF) of *T. b. rhodesiense* patients in Northern Uganda (35). These libraries were prepared conventionally after either rRNA-depletion for blood or poly-A selection for CSF samples. We analyzed only those samples for which at least 10% of reads mapped to the T. brucei genome. Raw reads from these samples were subjected to the VSG-seq analysis pipeline. Because the parasitemia of these patients was much higher than in our T. b. gambiense study, we adjusted our expression criteria accordingly to ≥0.01%, the published limit of detection of VSG-seq (25). Using this approach, we identified 77 unique *VSG* sequences across all blood and CSF samples (Fig. 3A; see also Fig. S6 in the supplemental material). SRA, the VSG-like protein that confers human serum resistance in *T. b. rhodesiense* (36), was detected in all patient samples.

10.1128/mbio.02553-22.6 FIG S6 Heatmap of all assembled *T. b. rhodesiense* patient VSGs. Greyscale shows log_10_ of the estimated percentage of the parasite population expressing each VSG. Variants expressed by less than 0.01% of parasites considered not detected (n.d.). Download FIG S6, EPS file, 1.1 MB. Copyright © 2022 So et al. 2022 So et al. https://creativecommons.org/licenses/by/4.0/ This content is distributed under the terms of the Creative Commons Attribution 4.0 International license.

The HMM pipeline determined types for 74 of these VSG sequences; the remaining sequences appeared to be incompletely assembled, presumably due to insufficient read depth from their low level of expression. Multiple *VSGs* assembled in each patient (Fig. 3A), and a large proportion of *VSGs* were expressed in multiple patients (Fig. 3C). Although most VSGs detected in these patients were type B (57%) (Fig. 3B), this VSG type was much less predominant than in T. b. gambiense infection. Interestingly, *T. b. rhodesiense* patient CSF revealed another possible layer of diversity in *VSG* expression, with 5 *VSGs* expressed exclusively in this space.

### The composition of the genomic VSG repertoire is reflected in expressed VSG N-terminal domain types.

**FIG 4:**
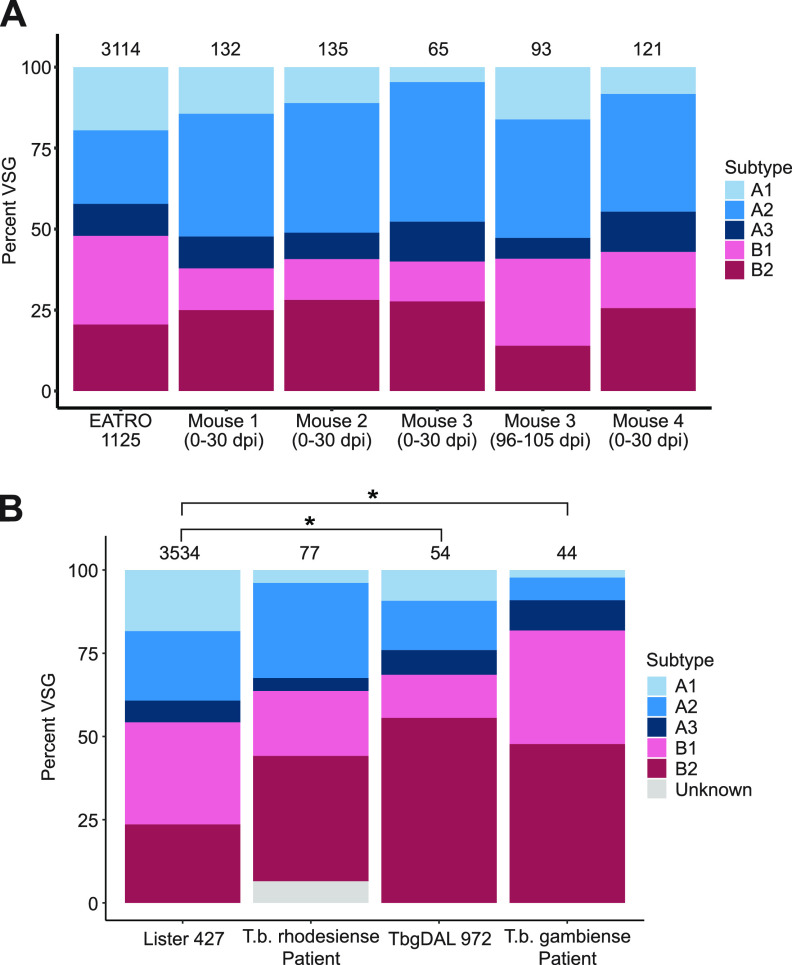
VSG expression reflects the genomic VSG repertoire of the infecting parasites. (A) Columns show the proportion of VSG types identified in each mouse infection over all time points and the proportion of VSG types in the infecting *T. b. brucei* strain, EATRO 1125. The total number of unique VSG sequences is displayed above each column. (B) A comparison of the frequencies of type A and B VSGs expressed in patients and those present in Lister 427 and DAL972 reference genomes. Relevant statistical comparisons are shown, and asterisks denote a *P* value of <0.05.

One source for bias in expressed VSG type is the composition of the genomic *VSG* repertoire. To investigate the relationship between expressed *VSG* repertoires and the underlying genome composition, we took advantage of our published VSG-seq analysis of parasites isolated from mice infected with the *T. b. brucei* EATRO1125 strain. As the “VSGnome” for this strain has been sequenced, we could directly compare the proportion of expressed N-terminal types to the full repertoire of types contained within the strain’s genome. In this experiment, blood was collected over time, providing data from days 6/7, 12, 14, 21, 24, 26, and 30 postinfection in all four mice and from days 96, 99, 102, and 105 in one of the four mice (mouse 3). Of 192 unique VSGs identified between days 0 and 30, the python HMM pipeline typed 190; of 97 unique VSGs identified between days 96 and 105, the pipeline typed 93 VSGs. The remaining VSGs were incompletely assembled by Trinity. Our analysis of VSG types over time revealed that the predominantly expressed N-terminal domain type fluctuates between type A and type B throughout the early stages of infection and in extended chronic infections (see Fig. S7 in the supplemental material), but the expressed *VSG* repertoire across all time points generally reflects the composition of the genomic repertoire (chi-squared *P* = 0.0515) (Fig. 4A). Parasitemia did not correlate with either the diversity of *VSG* expression or N-terminal domain type predominance (Fig. S2C).

10.1128/mbio.02553-22.7 FIG S7 VSG N-terminal type composition fluctuates over the course of infection in mice. Proportions of N-terminal domain types expressed in *T. b. brucei*-infected mice over time. The black dotted line represents the total number of identified VSGs. (A) N-terminal type composition days 0 to 30. (B) Type composition days 96 to 105. Download FIG S7, EPS file, 1.1 MB. Copyright © 2022 So et al. 2022 So et al. https://creativecommons.org/licenses/by/4.0/ This content is distributed under the terms of the Creative Commons Attribution 4.0 International license.

Unfortunately, the entire repertoire of *VSGs* encoded by most trypanosome strains is unknown, so such a direct comparison is impossible for T. b. gambiense and *T. b. rhodesiense* patient samples. Although the content of the “core” T. brucei genome (containing the diploid, housekeeping genes) is similar enough among subspecies for short-read resequencing projects to be scaffolded using the TREU927 or Lister 427 reference genomes (37–39), this method cannot be applied to investigate the *VSG* repertoires of subspecies (or even individual parasite strains [26]). Because no nearly complete VSGnome for any *T. b. rhodesiense* strain was available, we compared the makeup of *T. b. rhodesiense* expressed *VSGs* with the closely related and nearly complete *T. b. brucei* Lister 427 repertoire (38). We observed no difference in the proportions of N-terminal types (*P* = 0.2422, χ^2^ test) (Fig. 4B). Similarly, the proportion of N-terminal domains identified in the T. b. gambiense patient samples is not statistically different from the incomplete T. b. gambiense DAL972 genomic repertoire (*P* = 0.0575) (Fig. 4B). Both T. b. gambiense patient VSGs (*P* = 2.413e−4) and the 54 VSGs identified in T. b. gambiense DAL972 (*P* = 0.0301) have A and B type frequencies that differ significantly from the Lister427 genome. Overall, the frequency of each VSG N-terminal type is significantly different between all sources: *T. b. rhodesiense*, T. b. gambiense, and *T. b. brucei* all exhibit significantly different frequencies of expressed types (*P* = 1.492e−08), and the frequencies of encoded types in all three reference strains are significantly different from each other (*P* = 8.775e−08). We observe no statistical difference, however, between each expressed repertoire and the corresponding reference genome. Despite limitations in the available reference genomes, together these data support a model in which VSG types are drawn from the repertoire at a roughly equal frequency to their representation in the genome, with T. b. gambiense exhibiting an N-terminal type composition that differs from other subspecies.

### *VSGs* expressed by T. b. gambiense parasites are highly diverged from those found in the whole-genome sequences of other isolates.

We sought to understand how the *VSGs* expressed in the T. b. gambiense patient isolates related to known T. b. gambiense *VSG* sequences and whether there was evidence of recombination within the expressed *VSGs*. Initial attempts to BLAST the assembled *VSGs* against the DAL972 whole-genome assembly provided very few hits even using extremely permissive settings (-word_size 11 -evalue 0.1). This was unexpected but may reflect the relatively low coverage of the total VSG repertoire in the DAL972 genome assembly, which primarily covers the “core” genome.

To evaluate the relationship between the expressed *VSGs* and other isolates, we took advantage of publicly available short-read whole-genome data sets for 36 T. b. gambiense strains from the following three groups defined by their region and date of isolation: Côte d’Ivoire 1980s, Côte d’Ivoire 2000s, and DRC 2000s (40, 41). Because these are raw whole-genome data sets that have not undergone any assembly, they should include all VSG sequences, unlike DAL972. We searched for similarity between the expressed *VSGs* and each isolate genome by mapping short reads to each assembled expressed *VSG*: regions in which reads align to a specific VSG are present somewhere in the genome of the isolate, while regions with no alignments must either be unique to gHAT patients or sufficiently diverged to no longer map.

Using representative genes from the model organisms Caenorhabditis elegans, Drosophila melanogaster, and Escherichia coli as negative controls and T. b. gambiense glyceraldehyde-3-phosphate dehydrogenase (GAPDH) as a positive control, we determined the appropriate read length for evaluating sequence representation. The majority of each negative control gene (66.3% average across all controls) was covered by a successful alignment using 20-bp sequences and allowing 2 or fewer mismatches (see Fig. S8A in the supplemental material), indicating that read mapping at this length is not sufficiently specific. Increasing the sequence query length to 30 bp greatly decreased mapping to the negative controls, such that an average of 1.4% of each gene was represented within the genomic data sets. The T. b. gambiense GAPDH control, conversely, retained 100% read coverage across the whole gene at all read lengths (Fig. S8B). Thus, a 30-bp query is of appropriate stringency to measure the sequence representation of the patient *VSGs* within the whole-genome data sets.

10.1128/mbio.02553-22.8 FIG S8 Mapping controls show how read size affects stringency of alignments and support presence of sequences within data sets. (A) Base pair coordinates of bowtie alignment ranges using 20-bp read lengths and allowing 2 mismatches for each of the 36 whole-genome data sets. Positions of alignment hits are shown on the *x* axis, and each facet shows results for the 9 negative controls as well as 3 T. b. gambiense gene-positive controls. The negative controls are randomly selected genes from other model organisms. (B) Base pair coordinates for the same set of positive and negative gene mapping controls using 30-bp read lengths and allowing 2 mismatches. Coverage of the negative control genes is greatly reduced, while the T. b. gambiense gene-positive controls still have alignment hits across the entirety of the gene. Download FIG S8, TIF file, 1.1 MB. Copyright © 2022 So et al. 2022 So et al. https://creativecommons.org/licenses/by/4.0/ This content is distributed under the terms of the Creative Commons Attribution 4.0 International license.

**FIG 5:**
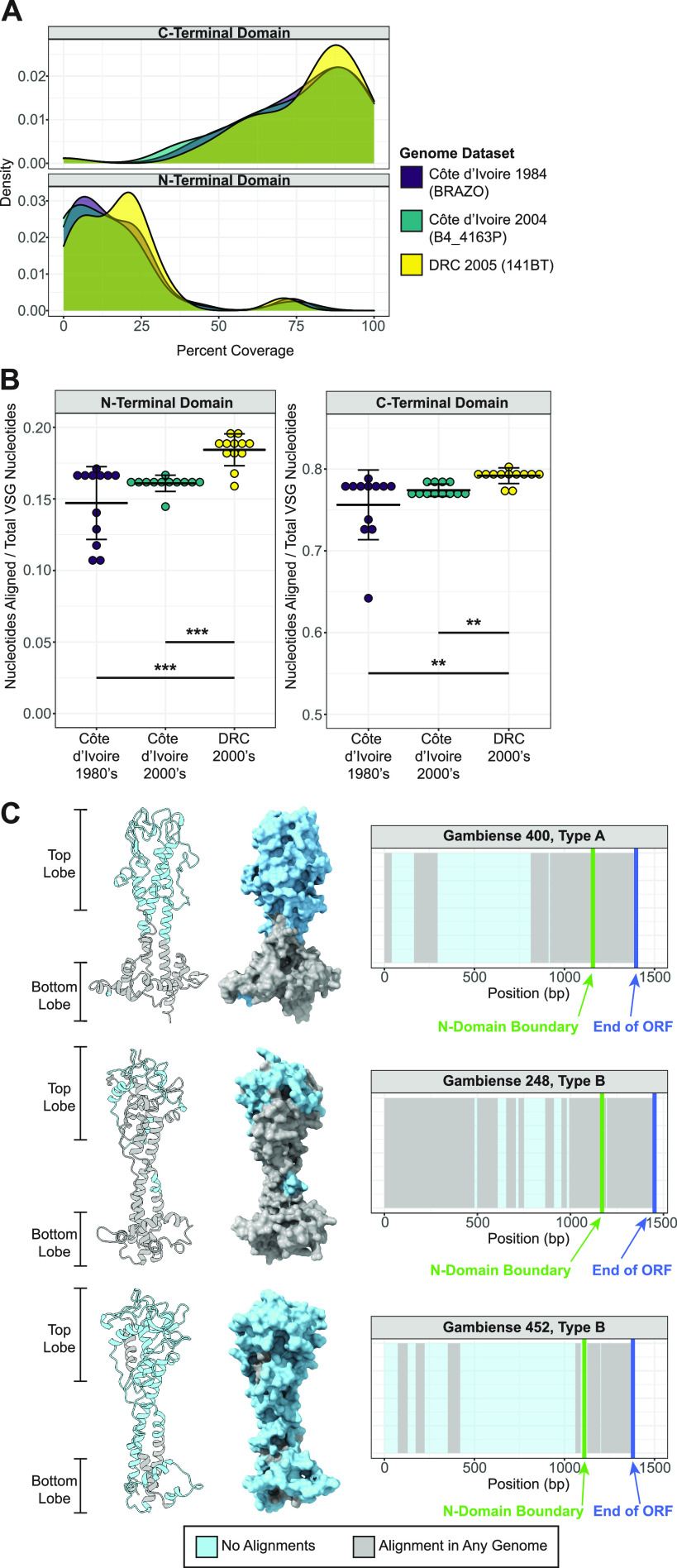
Diversification is most dramatic in exposed regions of the VSG. (A) Density plot showing the percentage of each of the patient VSG ORF sequence that had at least one whole-genome sequencing read (30-bp length) align for each of three representative whole-genome data sets (*n* = 12 per group). (B) Plots comparing sequence representation within the patient VSG N-terminal and C-terminal domains for each group. Representation for each VSG is quantified as the proportion of nucleotides in each domain with at least one alignment to the total number of nucleotides in that domain, with the average representation of all VSGs for each genome shown. Crossbars indicate mean and standard deviation within group. Significant differences between groups were determined using Kruskal-Wallis followed by a *post hoc* Dunn’s test (**, *P* < 0.01; ***, *P* < 0.001). (C) Models showing the predicted N-terminal domain structures of the three patient VSGs. The VSGs shown are the type A (Gambiense 248) and type B (Gambiense 452) VSGs with the highest reported ORF coverage and a type B VSG (Gambiense 452) with average ORF coverage. Monomer structures are oriented so the polymerization interface is away from the viewer. To the right of each model is a map of coverage across each *VSG* ORF. Regions with at least one alignment from any of the 36 genomic data sets are shown in gray, and regions with no alignment are shown in blue.

Using this query length, ~70% of the patient VSG open reading frame (ORF) on average was absent from each genome data set (see Fig. S9 in the supplemental material). Further analysis showed that C-terminal domain sequences were well represented within all genomic data sets regardless of origin (mean mapped read coverage = 77.4%), while there was relatively little nucleotide sequence similarity between the isolate genomes and the N termini expressed by parasites in gHAT patients (16.4%) (Fig. 5A). Aligned nucleotide coverage was significantly higher for the genomic data sets from strains also isolated in the DRC (where the gHAT patients originated) than those isolated in Côte d’Ivoire from either time period (Fig. 5B), suggesting a geographic component to *VSG* repertoires. Nonetheless, nucleotide coverage was still very low for DRC isolates when mapping to expressed N termini (18.4%) with no expressed VSG entirely present within the genomic data sets.

10.1128/mbio.02553-22.9 FIG S9 Summary of Bowtie alignment hits for each assembled gHAT patient VSG against the genomic sequences. (A) Base-pair coordinates of each patient VSG are plotted as the *x* axis, and each facet designates the patient VSG as well as the full ORF sequence length. Bars color-coded by genome dataset group show alignment length and position within the VSG ORF sequence for genomic sequence fragments of 30 bp in length. Download FIG S9, EPS file, 1.4 MB. Copyright © 2022 So et al. 2022 So et al. https://creativecommons.org/licenses/by/4.0/ This content is distributed under the terms of the Creative Commons Attribution 4.0 International license.

To understand where diverged sequences occurred on the VSG protein, we modeled the regions of sequence divergence on predicted N-terminal domain monomer structures of each patient VSG. Strikingly, we found that the DNA sequences that encoded residues in the top lobe of the protein were invariably absent from all genomic data sets (Fig. 5C). Overall, this analysis indicates that the *VSGs* expressed in the T. b. gambiense patient isolates are highly diverged from those within the DAL972 genome as well as from other sequenced field isolates, particularly within the parts of N-terminal domain most likely to interface with host antibody. These results are also consistent with geographic variation in T. b. gambiense *VSG* repertoires.

## DISCUSSION

African trypanosomes evade the host adaptive immune response through a process of antigenic variation where parasites switch their expressed *VSG* (42). The genome of T. brucei encodes a large repertoire of *VSG* genes, pseudogenes, and gene fragments that can be expanded continuously through recombination to form entirely novel “mosaic” *VSGs* (16). While antigenic variation has been studied extensively in culture and animal infection models, our understanding of the process in natural infections, particularly human infection, is limited. Most experimental mouse infections are sustained for weeks to months, while humans and large mammals may be infected for several months or even years. Additionally, laboratory studies of antigenic variation almost exclusively use *T. b. brucei*, a subspecies of T. brucei that, by definition, does not infect humans. The primary hurdle to exploring antigenic variation in nature has been technical: it is difficult to obtain sufficient parasite material for analysis. This is especially true for infection with T. b. gambiense, which often exhibits extremely low parasitemia. Here, we have demonstrated the feasibility of VSG-seq to analyze *VSG* expression in RNA samples isolated directly from HAT patients. Our analyses reveal unique aspects of antigenic variation in T. b. gambiense that can only be explored by studying natural infections.

We have identified an intriguing bias toward the expression of type B VSGs in T. b. gambiense infection, which appears to be specific to this T. brucei subspecies. Comparison of expressed *VSG* repertoires to publicly available genomic *VSG* repertoires suggests that the genomic *VSG* repertoire determines the distribution of VSG N-terminal types expressed during T. brucei infection. Thus, the T. b. gambiense *VSG* repertoire may contain a larger proportion of type B VSGs than its more virulent counterparts. Could a bias toward certain VSG types, whether due to a difference in repertoire composition or expression preference, account for unique features of T. b. gambiense infection, including its chronicity and primarily anthroponotic nature (43)?

Little is known about how differences in VSG proteins relate to parasite biology or whether there could be biological consequences to the expression of specific VSG N- or C-terminal types. Type A *var* genes in Plasmodium falciparum infection are associated with severe malaria (44–48), and similar mechanisms have been hypothesized to exist in Trypanosoma vivax and Trypanosoma congolense infections (49–52). In T. brucei, several VSGs have evolved specific functions beside antigenic variation (52). The first type B VSG structure was recently solved (53), revealing a unique *O*-linked carbohydrate in the VSG’s N-terminal domain that interfered with the generation of protective immunity in a mouse infection model. Perhaps structural differences between each VSG type, including glycosylation patterns, could influence infection outcomes. Further research will be needed to determine whether the observed predominance of type B VSGs could influence the biology of T. b. gambiense infection.

Another possibility that we cannot rule out, however, is that the gHAT samples are biased due to selection by the serological test used for diagnosis. Patients were screened for T. b. gambiense infection using the CATT, a serological test that uses parasites expressing VSG LiTat 1.3 as an antigen. LiTat 1.3 contains a type B2 N-terminal domain (54, 55). Patients infected with parasites predominantly expressing type B VSGs may be more likely to generate antibodies that cross-react with LiTat 1.3, resulting in preferential detection of these cases. In contrast, *T. b. rhodesiense* can only be diagnosed microscopically, removing the potential to introduce bias through screening. It remains to be investigated whether samples from patients diagnosed using newer screening tests, which include the invariant surface glycoprotein ISG65 and the type A VSG LiTat 1.5 (22), would show similar bias toward the expression of type B VSGs.

Such a bias, if it exists, would be important to understand, as it could affect the ability to detect a subset of gHAT infections. The diversity and corresponding divergence of expressed VSGs from publicly available genomic sequences could have similar implications. Although diversity in T. b. gambiense infection appeared lower overall than previous measurements from experimental mouse infections (16, 17, 25), the correlation that we observed between parasitemia and diversity in T. b. gambiense isolates suggests that our sampling was incomplete. Indeed, in our analysis of *T. b. rhodesiense* infection (a more reasonable comparison to mouse infection given similar expression cutoffs and parasitemia), we observed diversity similar to or higher than what has been observed in *T. b. brucei* mouse infections. Moreover, *T. b. rhodesiense* patient CSF revealed another layer of diversity in *VSG* expression, with 5 *VSGs* expressed exclusively in this space. Although this observation is difficult to interpret without information about the precise timing of sample collection, a recent study in mice showed that extravascular spaces harbor much of the antigenic diversity during infection (56). It is exciting to speculate that different organs or body compartments could harbor different sets of VSGs in humans as well.

Overall, our analysis of *VSG* expression in T. b. gambiense and *T. b. rhodesiense* patients confirmed the long-held assumption that *VSG* diversity is a feature of natural infection. One potential consequence of this striking diversity is that the genomic *VSG* repertoire might be exploited very rapidly, creating pressure for the parasite to diversify its *VSG* repertoire as the mammalian host generates antibodies against each expressed *VSG*. Our results are consistent with this, revealing extreme divergence in the patient *VSGs* from 36 publicly available T. b. gambiense whole-genome sequencing data sets. Even when mapping relatively short 30-bp genomic sequences to each VSG, we could only find evidence for ~30% of each *VSG* ORF. Without assembled genomes, it is difficult to infer recombination patterns or mechanisms from this analysis. The fact that only very short stretches of homology could be found within the N-terminal domain, however, is consistent with recombination through microhomology-mediated end joining, a DNA repair mechanism that uses short stretches of homology (5 to 20 bp) to repair DNA damage (57). This appears to be the favored form of DNA repair in the *VSG* expression site and has been hypothesized to play a role in *VSG* switching (57, 58). The data presented here suggest this mechanism, or a similar one, may play a role in diversification of the *VSG* repertoire as well.

We also observed divergence between geographically separate parasite populations. Past research has shown that the sensitivity of serological tests for gHAT, which detect antibodies against the LiTat 1.3 VSG, vary regionally, potentially due to differences in the underlying genomic or expressed *VSG* repertoire in circulating strains (54, 55). Our data are consistent with such a possibility, with the *VSGs* expressed in patients from the DRC sharing more sequence similarity with isolates from the same country than those from Côte d’Ivoire. Geographic variation has been observed in *var* gene repertoires of Plasmodium falciparum (59) and the *VSG* repertoire of *Trypanosoma vivax*, another African trypanosome (51). A better understanding of such differences in T. brucei could inform the development of future HAT diagnostics.

The positions of divergent regions within the VSG protein demonstrate the enormous pressure exerted by host antibody on the repertoire of T. b. gambiense. While the C termini of patient *VSGs* were well-represented, the majority of each N-terminal sequence was undetectable in the 36 genomes that we analyzed. Notably, in even the most conserved VSG N termini, sequences encoding the top lobe of the VSG were completely absent from the genomes that we analyzed. VSG proteins are packed together very closely on the parasite cell surface, presumably preventing host antibody from accessing epitopes close to or within the C terminus (32). Thus, those regions with no nucleotide similarity correspond directly to the parts of the VSG protein most likely to be exposed to host antibody.

In addition to confirming that certain aspects of antigenic variation observed in experimental T. brucei infection are features of natural infection, this study has revealed unique features of the process in T. b. gambiense. This subspecies appears to preferentially express certain VSG N termini, which could be related to the unique biology of the parasite. Additionally, wild *VSG* repertoires may be more diverse than previously expected with potential geographic variation. While mouse models can recapitulate certain aspects of the process, new biology remains to be uncovered by studying antigenic variation in its natural context.

## MATERIALS AND METHODS

### Ethics statement.

The blood specimens from T. b. gambiense-infected patients were collected within the projects, “Longitudinal follow-up of CATT seropositive, trypanosome negative individuals (SeroSui)” and “An integrated approach for identification of genetic determinants for susceptibility for trypanosomiasis (TrypanoGEN)” (60). In France, the SeroSui study received approval from the Comité Consultatif de Déontologie et d’Ethique (CCDE) of the French National Institute for Sustainable Development Research (IRD), May 2013 session. In Belgium, the study received approval from the Institutional Review Board of the Institute of Tropical Medicine (reference 886/13) and the Ethics Committee of the University of Antwerp (B300201318039). In the Democratic Republic of the Congo, the projects SeroSui and TrypanoGEN were approved by the Ministry of Health through the Ngaliema Clinic of Kinshasa (references 422/2013 and 424/2013). Participants gave their written informed consent to participate in the projects. For minors, additional written consent was obtained from their legal representative.

### Patient enrollment and origin map.

Patients originated from the DRC and were identified over 6 months in the second half of 2013. This identification occurred either during passive screening at the center for HAT diagnosis and treatment at the hospital of Masi Manimba or during active screening by the mobile team of the national sleeping sickness control program (PNLTHA) in Masi Manimba and Mosango health zones (Kwilu province, DRC).

Individuals were screened for the presence of specific antibodies in whole blood with the CATT test. For those reacting blood positive in CATT, we also tested 2-fold serial plasma dilutions of 1/2 to 1/32 and determined the CATT end titer. CATT positives underwent parasitological confirmation by direct microscopic examination of lymph (if enlarged lymph nodes were present) and examination of blood by the mini-anion exchange centrifugation technique on buffy coat (61). Individuals in whom trypanosomes were observed underwent lumbar puncture. The cerebrospinal fluid was examined for white blood cell count and the presence of trypanosomes to determine the disease stage and select the appropriate treatment. Patients were questioned about their place of residence. The geographic coordinates of their corresponding villages were obtained from the Atlas of HAT (62) and plotted on a map created using ArcGIS software by Esri. ArcGIS is the intellectual property of Esri and is used herein under license. Copyright Esri. All rights reserved. Distances were determined and a distance matrix generated (see matrix at https://github.com/mugnierlab/Tbgambiense2021/).

### Patient blood sample collection and total RNA isolation.

A 2.5-mL volume of blood was collected from each patient in a PAXgene blood RNA tube. The blood was mixed with the buffer in the tube, aliquoted in 2-mL volumes, and frozen in liquid nitrogen for a maximum of 2 weeks. After arrival in Kinshasa, tubes were stored at −70°C. Total RNA was extracted and isolated from each blood sample as previously described (27).

### Estimation of parasitemia.

Two approaches were used to estimate parasitemia. First, a 9-mL volume of blood on heparin was centrifuged, 500 μL of the buffy coat were taken up, and trypanosomes were isolated using the mini-anion exchange centrifugation technique. After centrifugation of the column eluate, the number of parasites visible in the tip of the collection tube were estimated. Second, spliced leader (SL) RNA expression levels were measured by real-time PCR as previously described (27). A threshold cycle (*C_T_*) value was determined for each patient blood sample. Real-time PCR was performed on RNA samples before reverse transcription to verify the absence of DNA contamination.

### RNA sequencing.

DNase I-treated RNA samples were cleaned up with 1.8× Mag-Bind total pure NGS beads (Omega Bio-Tek; number M1378-01). cDNA was generated using the SuperScript III first-strand synthesis system (Invitrogen; number 18080051) according to manufacturer’s instructions. Eight microliters of each sample (between 36 and 944 ng) was used for cDNA synthesis, which was performed using the oligo-dT primer provided with the kit. This material was cleaned up with 1.8× Mag-Bind beads and used to generate three replicate library preparations for each sample. These technical replicates were generated to ensure that any *VSGs* detected were not the result of PCR artifacts (63, 64).

Because we expected a low number of parasites in each sample, we used a nested PCR approach to prepare the VSG-seq libraries. First, we amplified T. brucei cDNA from the parasite/host cDNA pool by PCR using a spliced leader primer paired with an anchored oligo-dT primer (SL-1-nested and anchored oligo-dT). Twenty cycles of PCR were completed (55°C annealing, 45 s extension) using Phusion polymerase (Thermo Scientific; number F530L). PCRs were cleaned up with 1.8× Mag-Bind beads. After amplifying T. brucei cDNA, a *VSG*-specific PCR was carried out using M13RSL and 14-mer-SP6 primers (see primers at https://github.com/mugnierlab/Tbgambiense2021/). Thirty cycles of PCR (42°C annealing, 45 s extension) were performed using Phusion polymerase. Amplified *VSG* cDNA was then cleaned up with 1× Mag-Bind beads and quantified using a Qubit double-stranded DNA (dsDNA) HS assay (Invitrogen; number Q32854).

Sequencing libraries were prepared from 1 ng of each VSG PCR product using the Nextera XT DNA library preparation kit (Illumina; number FC-131-1096) following the manufacturer’s protocol except for the final cleanup step, which was performed using 1× Mag-Bind beads. Single-end 100-bp sequencing was performed on an Illumina HiSeq 2500.

### VSG-seq analysis of T. b. gambiense and *T. b. rhodesiense* sequencing libraries.

To analyze both T. b. gambiense (VSG-seq preparations) and *T. b. rhodesiense* (traditional mRNA sequencing library preparations; sequences were obtained from ENA, accession numbers PRJEB27207 and PRJEB18523), we processed raw reads using the VSG-seq pipeline available at https://github.com/mugnierlab/VSGSeqPipeline. Briefly, *VSG* transcripts were assembled *de novo* from quality- and adapter-trimmed reads for each sample (patient or patient replicate) from raw reads using Trinity (version 5.26.2) (65). Contigs containing open reading frames (ORFs) were identified as previously described (25). ORF-containing contigs were compared to Lister 427 and EATRO1125 *VSGs* as well as a collection of known contaminating non-*VSG* sequences. Alignments to *VSGs* with an E value below 1 × 10^−10^ that did not match any known non-*VSG* contaminants were identified as *VSG* transcripts. For T. b. gambiense replicate libraries, *VSG* ORFs identified in any patient replicate were consolidated into a sole reference genome for each patient using CD-HIT (version 4.8.1) (66) with the following arguments: -d 0 -c 0.98 -n 8 -G 1 -g 1 -s 0.0 -aL 0.0. Final *VSG* ORF files were manually inspected.

Two T. b. gambiense patient *VSG*s (patients 11 and 13) showed likely assembly errors. In one case, a *VSG* was duplicated and concatenated, and in another, two *VSGs* were concatenated. These reference files were manually corrected (removing the duplicate or editing annotation to reflect two *VSGs* in the concatenated ORF) so that each *VSG* could be properly quantified. For T. b. gambiense, we then aligned reads from each patient replicate to that patient’s consolidated reference genome using Bowtie with the parameters *-v 2 -m 1 -S* (version 1.2.3) (67).

For *T. b. rhodesiense*, we aligned each patient’s data to its own *VSG* ORF assembly. Reads per kilobase per million (RPKM) values for each *VSG* in each sample were generated using MULTo (version 1.0) (68), and the percentage of parasites in each population expressing a *VSG* was calculated as described previously (25). For T. b. gambiense samples, we included only *VSGs* with an expression measurement above 1% in two or more patient replicates in our analysis. For *T. b. rhodesiense* samples, we included only *VSGs* with expression >0.01%. To compare *VSG* expression between patients, despite the different reference genomes used for each patient, we used CD-HIT to cluster *VSG* sequences with greater than 98% similarity among patients, using the same parameters used to consolidate reference *VSG* databases before alignment. We gave each unique *VSG* cluster a numerical ID (e.g., Gambiense number) and chose the longest sequence within each group to represent the cluster. Before analysis, we manually removed clusters representing TgsGP and SRA from the expressed *VSG* sets. UpSet plots were made with the UpSetR package (69). *VSG* reference databases for each patient as well as the R code used to analyze resulting data is available at https://github.com/mugnierlab/Tbgambiense2021/.

### Analysis of VSG N-terminal domains.

**(i) Genomic *VSG* sequences.** The *VSG* repertoires of *T. b. brucei* Lister 427 (“Lister427_2018” assembly), *T. b. brucei* TREU927/4, and T. b. gambiense DAL972 were taken from TriTrypDB (v50), while the *T. b. brucei* EATRO1125 VSGnome was used for analysis of the EATRO1125 *VSG* repertoire (vsgs_tb1125_nodups_atleast250aas_pro.txt, available under GenBank accession numbers KX698609.1 to KX701858.1 or https://tryps.rockefeller.edu/Sequences.html) . VSG sequences from other strains (except those generated by VSG-seq) were taken from the analysis in Cross et al. (14). Likely VSG N termini were identified as predicted proteins with significant similarity (E value ≤ 10^−5^) to hidden Markov models (HMMs) of aligned type A and B VSG N termini taken from reference 14.

**(ii) N-terminal domain phylogenies.** Phylogenies of VSG N termini based on unaligned sequence similarities were constructed using the method described in reference 70 and used previously to classify VSG sequence (14). We extracted predicted N-terminal domain protein sequences by using the largest bounding envelope coordinates of a match to either type A or type B HMM. A matrix of similarities between all sequences was constructed from normalized transformed BLASTp scores as in Wickstead and Gull (70) and used to infer a neighbor-joining tree using QuickTree v1.1 (71). Trees were annotated and visualized in R with the package APE v5.2 (72).

**(iii) HMM.** For N-terminal typing by HMM, we used a python analysis pipeline available at (https://github.com/mugnierlab/find_VSG_Ndomains). The pipeline first identifies the boundaries of the VSG N-terminal domain using the type A and type B HMM profiles generated by Cross et al., which includes 735 previously-typed VSG N-terminal domain sequences (14). N-terminal domains are defined by the largest envelope domain coordinate that meets E value threshold (1 × 10^−5^, –domE 0.00001). In cases where no N-terminal domain is identified using these profiles, the pipeline executes a “rescue” domain search in which the VSG is searched against a “pan-VSG” N terminus profile that we generated using 763 previously-typed VSG N-terminal domain sequences. This set of VSGs includes several T. brucei strains and/or subspecies as follows: Tb427 (559), TREU927 (138), T. b. gambiense DAL972 (28), EATRO795 (8), EATRO110 (5), Trypanosoma equiperdum (4), and Trypanosoma evansi (21). The N-terminal domain type of these VSGs was previously determined by Cross et al. by building neighbor-joining trees using local alignment scores from all-versus-all BLASTp similarity searches (14). Domain boundaries are called using the same parameters as with the type A and B profiles.

After identifying boundaries, the pipeline extracts the sequence of the N-terminal domain, and this is searched against five subtype HMM profiles. To generate N-terminal domain subtype HMM profiles, five multiple sequence alignments were performed using Clustal Omega (73) with the 763 previously-typed VSG N-terminal domain sequences described above; each alignment included the VSG N-terminal domains of the same subtype (A1, A2, A3, B1, and B2). Alignment output files in STOCKHOLM format were used to generate distinct HMM profiles for type A1, A2, A3, B1, and B2 VSGs using the predetermined subtype classifications of the 763 VSGs using HMMer version 3.1b2 (74). The number of sequences used to create each subtype profile ranged from 75 to 211. The most probable subtype is determined by the pipeline based on the highest scoring sequence alignment against the subtype HMM profile database when HMMscan is run under default alignment parameters. The pipeline generates a FASTA file containing the amino acid sequence of each VSG N terminus and a Comma separated values (CSV) with descriptions of the N-terminal domain including its type and subtype.

**(iv) Network graph.** N-terminal network graphs were made using VSG N-terminal domains from the TriTrypDB Lister427_2018 and T. b. gambiense DAL972 (v50) VSG sets described above and the T. b. gambiense and *T. b. rhodesiense* patient VSG N termini, which met our expression thresholds. Identified N-terminal domains were then subjected to an all-versus-all BLASTp. A pairwise table was created that includes each query-subject pair, the corresponding alignment E-value, and N-terminal domain type of the query sequence if previously typed in Cross et al. (14). Pseudogenes and fragments were excluded from the Lister427_2018 reference prior to plotting by filtering for *VSG* genes annotated as pseudogenes and any less than 400 amino acids in length, as the remaining sequences are most likely to be full-length VSG. Network graphs were generated with the igraph R package (75) using undirected and unweighted clustering of nodes after applying link cutoffs based on an E value of <10^−2^. The leading eigenvector clustering method (34) was used to detect and assign nodes to communities based on clustering (cluster_leading_eigen() method in igraph).

### Analysis of VSG C-terminal domains.

VSG C termini were extracted from expressed T. b. gambiense *VSGs*, T. b. gambiense DAL972 (v50), and 545 previously-typed VSG C termini from the Lister 427 strain using the C-terminal HMM profile generated by Cross et al. (14) and the same HMMscan parameters as for N termini (E value < 1 × 10^−5^; largest domain based on envelope coordinates). An all-versus-all BLASTp was performed on these sequences, and network graphs were generated in the same manner as the N-terminal network graphs. Links were drawn between C termini with a BLASTp E value of 1 × 10^−3^. The leading eigenvector method for clustering (34) was used to detect and assign nodes to communities based on clustering (cluster_leading_eigen() method in igraph).

### Comparison of gHAT patient VSGs to sequenced whole genomes of T. b. gambiense isolates.

Publicly available whole-genome Illumina sequencing reads for 24 T. b. gambiense isolates from Côte d’Ivoire were fetched from the ENA database, and 12 data sets for isolates from the DRC were downloaded from DataDryad. All data sets analyzed exist as raw sequencing reads and do not have associated ORF assemblies or VSG gene annotations. We therefore determined the presence or absence of sequences similar to patient VSG by alignment. Raw reads were adapter and quality trimmed using Trim_Galore (version 0.5.0) under default parameters and truncated to desired query lengths of 20, 30, and 50 bp using Trimmomatic (76) (version 0.38) ‘CROP’ option. Whole-genome sequence data sets were aligned to the assembled patient VSG nucleotide sequences using Bowtie with the parameters *-v 2 -a -S* (version 1.1.1). Bowtie does not support gapped alignments, and the number of mismatched bases per read can be adjusted to control the stringency of alignments; therefore, this aligner was used to assess the size of regions of sequence similarity between the patient VSG and genomic sequences. Bedtools (77) (version 2.27.0) genomecov was used to summarize alignment coordinates and read depth for downstream analysis. Alignment ranges were plotted with the IRanges R package (78). Patient VSG gene coverage was calculated as the regions of sequence with an aligned read depth of at least one divided by the full ORF sequence or domain length in base pairs.

To model regions of sequence divergence and similarity, the secondary structures for each of the 44 gHAT patient VSG were predicted using Phyre2 (79) batch processing under default parameters. Automated threading returned hits to VSG N-terminal domain chain templates from the PDB with 100% confidence for all patient VSG. Predicted structures were visualized and figures generated in ChimeraX (80).

### Data availability.

Raw data are available in the National Center for Biotechnology Information (NCBI) Sequence Read Archive under accession number PRJNA751607. Additional supplemental data sets are accessible at https://github.com/mugnierlab/Tbgambiense2021/.
