# Supplementary figures and images for: VSGs Expressed during Natural T. b. gambiense Infection Exhibit Extensive Sequence Divergence and a Subspecies-Specific Bias towards Type B N-Terminal Domains
Source: mBio. 2022 Nov 10;13(6):e02553-22. doi: 10.1128/mbio.02553-22 (PMC9765701; doi:10.1128/mbio.02553-22)

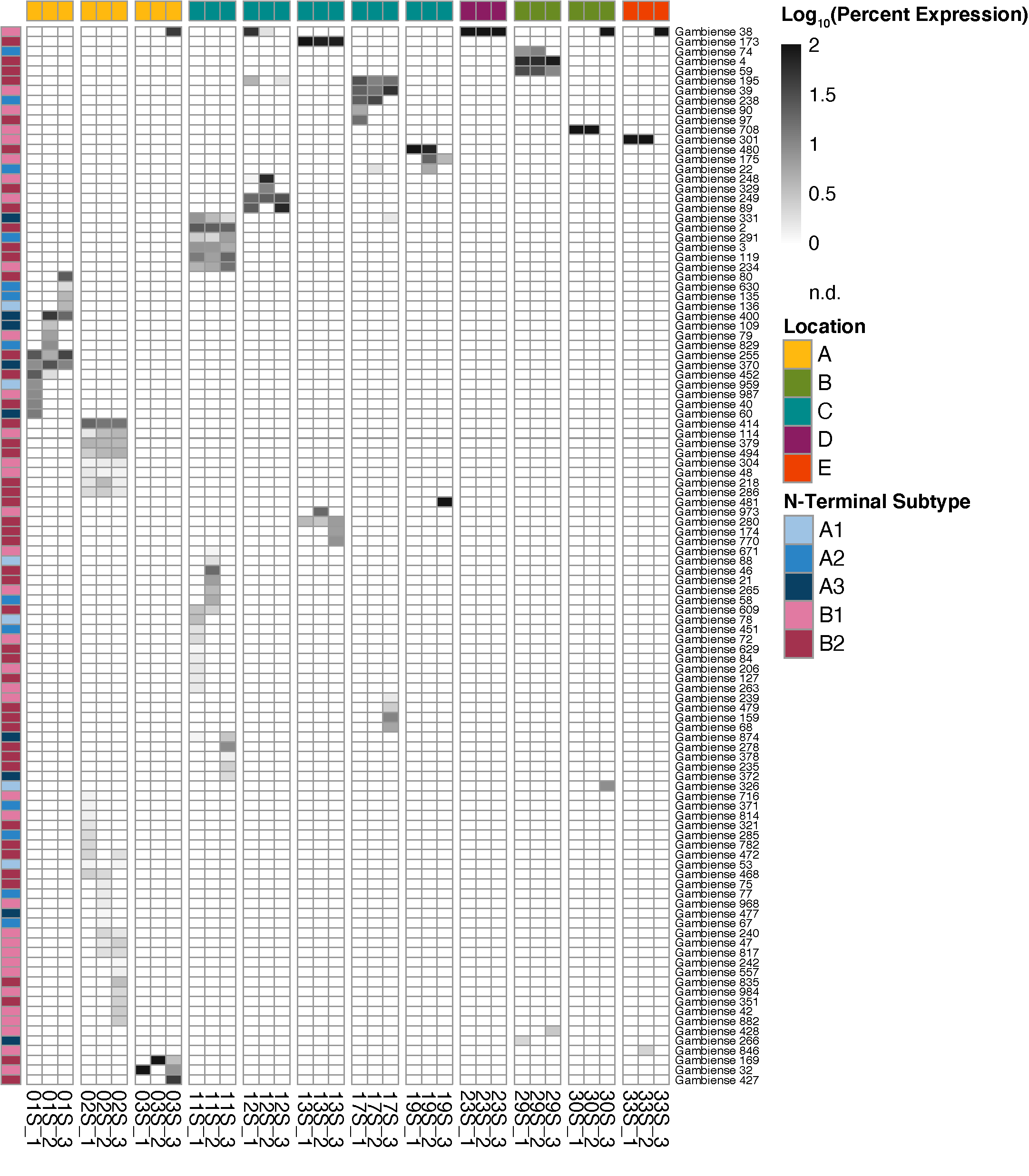

Supplement: FIG S1 [file mbio.02553-22-s0001.tif]

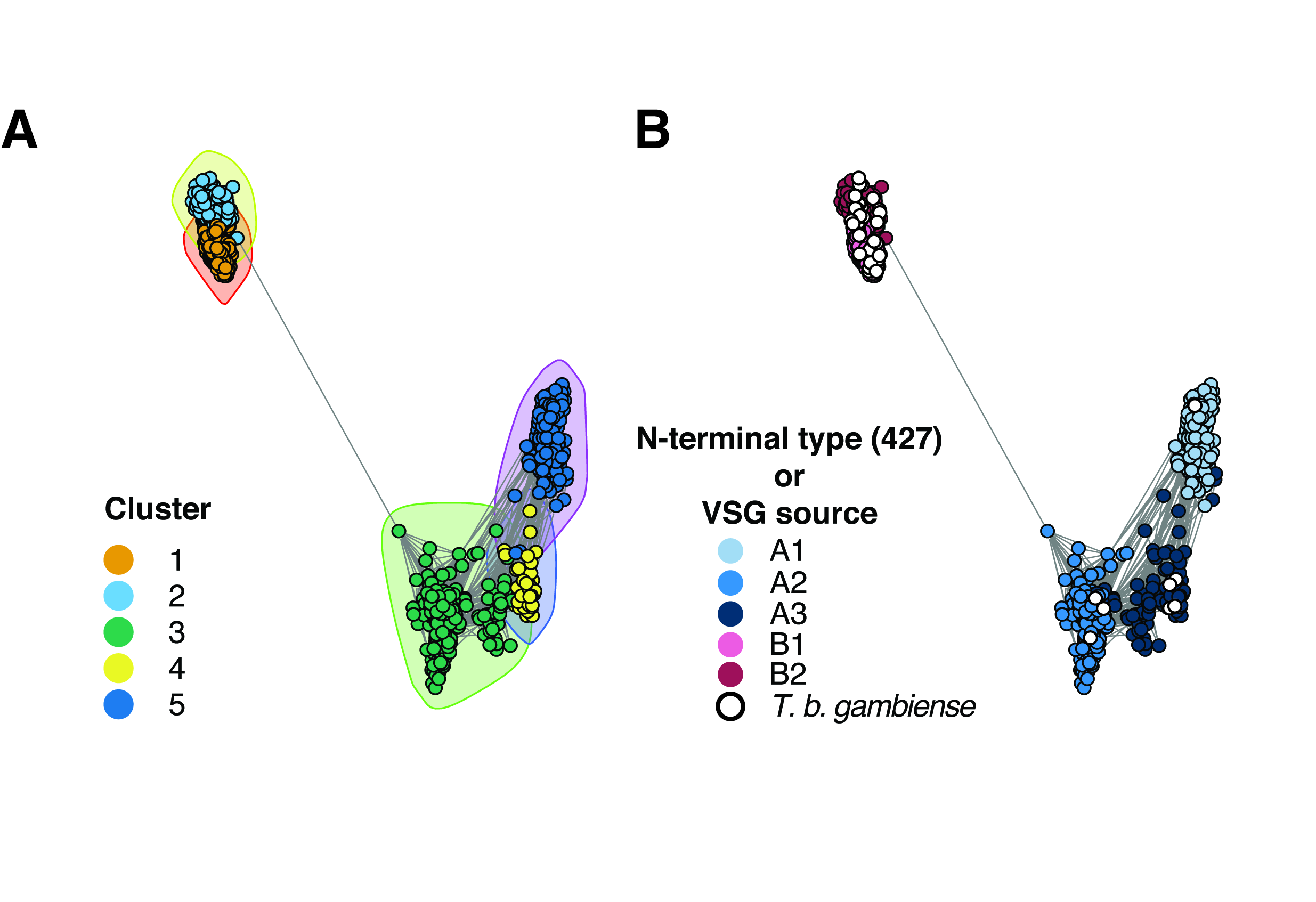

Supplement: FIG S3 [file mbio.02553-22-s0003.tif]

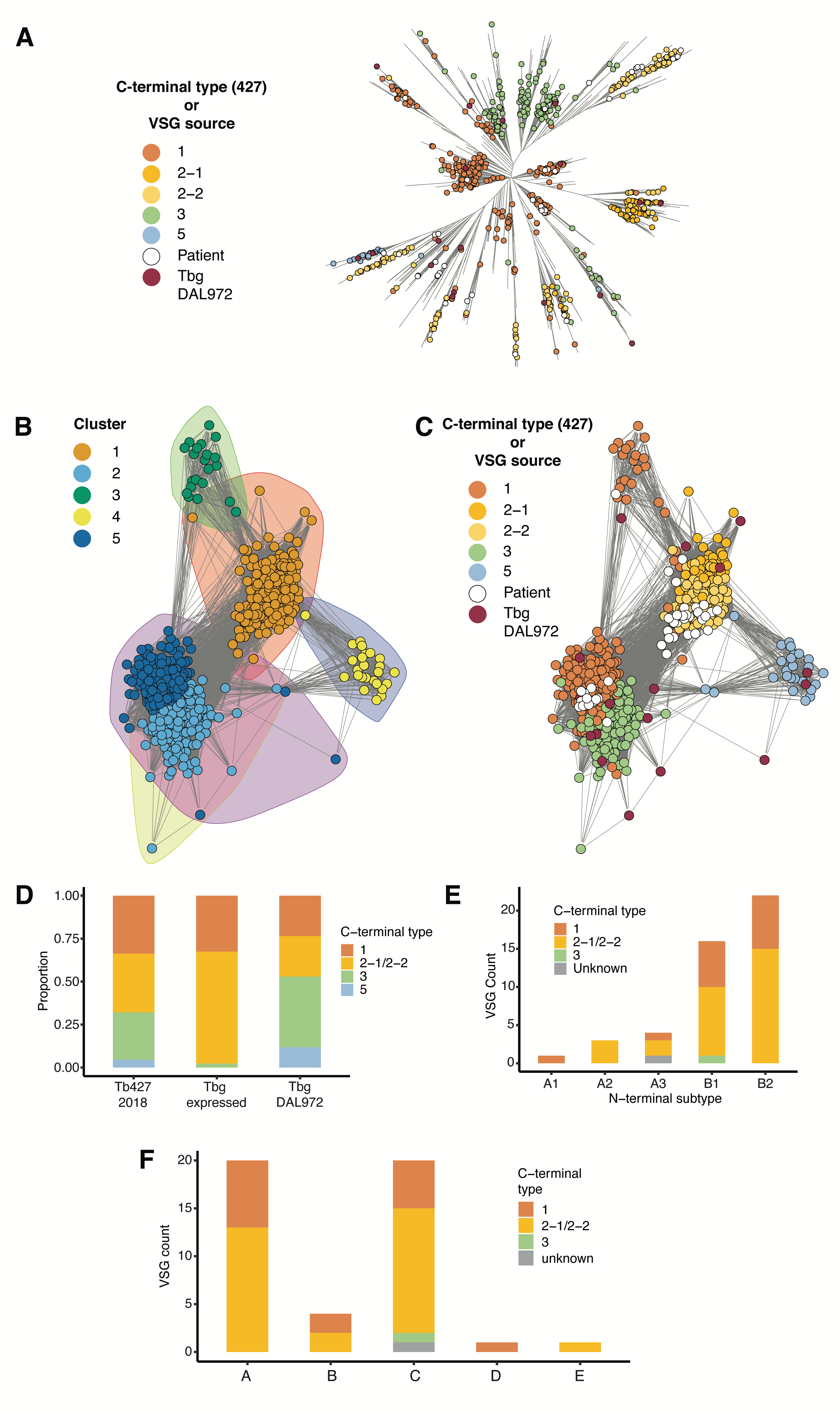

Supplement: FIG S5 [file mbio.02553-22-s0005.tif]

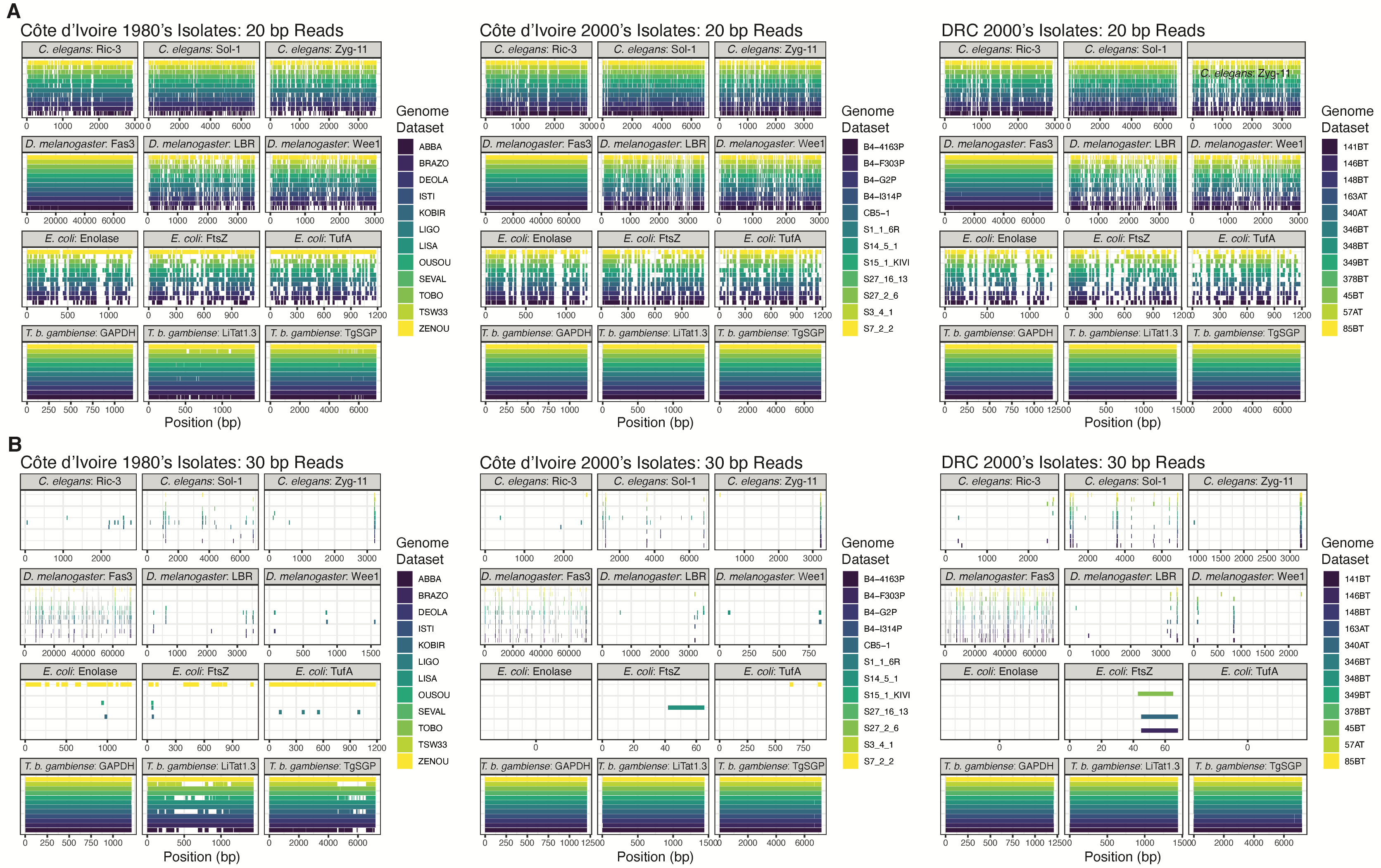

Supplement: FIG S8 [file mbio.02553-22-s0008.tif]
